# Pioneering Respiratory and Emergency Medicine Research During the COVID-19 Era

**DOI:** 10.3390/jpm15020073

**Published:** 2025-02-18

**Authors:** Ioannis Pantazopoulos, Georgios Mavrovounis, Konstantinos Gourgoulianis

**Affiliations:** 1 Department of Emergency Medicine, Faculty of Medicine, University of Thessaly, Biopolis, 41500 Larissa, Greece; mavrovou@uth.gr; 2 Department of Respiratory Medicine, Faculty of Medicine, University of Thessaly, Biopolis, 41500 Larissa, Greece; kgourg@uth.gr

## 1. Introduction

Respiratory and emergency medicine have undergone significant transformations in recent years, shaped by advancements in critical care, the integration of artificial intelligence (AI), and the evolving understanding of post-infectious sequelae. The COVID-19 pandemic accelerated research in several fields, leading to novel therapeutic and prognostic strategies. Simultaneously, emergency medicine has witnessed progress in early risk stratification, metabolic assessment, and non-invasive interventions, improving patient outcomes in both acute and chronic conditions. Despite these strides, significant knowledge gaps remain. The real-world implementation of AI-driven prognostic models remains limited due to concerns regarding their reliability and integration into clinical workflows [1]. The long-term impact of COVID-19 on respiratory function is still incompletely understood, particularly in patients with pre-existing lung diseases [2,3]. Furthermore, the disruption of routine healthcare during the pandemic has affected the management of chronic respiratory diseases, underscoring the need for alternative care delivery models [4].

This Special Issue of Journal of Personalized Medicine brings together a diverse collection of studies addressing these critical challenges. The contributions explore novel prognostic models, innovative therapeutic approaches, and emerging diagnostic techniques, offering new insights into the future of respiratory and emergency medicine. By bridging these knowledge gaps, the research presented herein provides a foundation for further investigations and clinical advancements in the field.

## 2. Prognostic Tools and Risk Stratification in Critical Care

Accurate prognostication in critically ill patients remains a cornerstone of emergency and intensive care medicine. Traditional scoring systems, such as the Sequential Organ Failure Assessment (SOFA) score, have been widely used to predict mortality in sepsis and COVID-19 patients [5]. However, recent advancements in machine learning-based risk models have shown promise in enhancing the predictive accuracy of clinical deterioration, particularly in mechanically ventilated patients [6]. Despite these advances, challenges remain in ensuring the generalizability of prognostic tools across diverse patient populations and clinical settings.

In this Special Issue, Hong et al. analyzed prognostic factors in elderly critically ill COVID-19 patients. Their multicenter cohort study, involving 434 patients, revealed that geriatric patients (aged ≥ 80 years) exhibited higher Sequential Organ Failure Assessment (SOFA) scores, indicating more severe organ dysfunction and higher mortality compared to younger elderly patients (aged 65–79 years), showing that renal function and prone positioning were key predictors of outcomes (Contribution 1).

Meanwhile, Chen et al. validated the MEDS and REMS scores for predicting mortality in patients with emphysematous cystitis, demonstrating how risk stratification models can be applied beyond respiratory diseases (Contribution 2).

In our opinion, future research should focus on integrating AI-driven predictive models into real-time ICU workflows for continuous patient monitoring while also ensuring that scoring systems are prospectively validated across diverse healthcare settings to improve their clinical utility.

## 3. Advances in Managing COVID-19 and Post-COVID-19 Complications

The long-term impact of COVID-19 on respiratory function remains an area of active investigation. Post-COVID-19 pulmonary and extrapulmonary sequelae and complications have become increasingly recognized, underscoring the need for post-discharge assessment [7,8]. Additionally, the potential for non-invasive interventions to reduce the viral load and mitigate the severity of symptoms in respiratory infections has received increasing interest, particularly explorations of their broader applications beyond COVID-19 [9].

Perroni et al. investigated the incidence of tracheal stenosis among ICU-hospitalized COVID-19 patients who underwent prolonged intubation or tracheostomy. The prospective multicenter study in Lombardy, Italy, demonstrated a notable incidence of tracheal lumen reduction, emphasizing the importance of long-term respiratory follow-up in post-COVID-19 care (Contribution 3). Regarding non-invasive interventions, Pantazopoulos et al. evaluated the effectiveness of nasal irrigation with a hypertonic seawater solution, demonstrating its potential as a low-cost, adjunctive therapy for respiratory infections beyond COVID-19. These findings highlight the importance of developing post-COVID-19 airway management protocols to prevent complications while also exploring non-invasive adjunct therapies as part of a broader respiratory care strategy (Contribution 4).

## 4. Metabolic and Physiological Insights in Critical Illness

The evaluation of metabolic disturbances and fluid management strategies remains a critical component of intensive care and emergency medicine. Traditional acid–base assessment methods have long been relied upon for guiding interventions, but more elaborate approaches such as the Stewart physicochemical method may offer improved diagnostic accuracy in detecting metabolic derangements [10]. However, despite its theoretical advantages, the Stewart method has seen limited clinical adoption, primarily due to unfamiliarity among practitioners and the absence of streamlined integration processes into routine practice.

Sotiropoulou et al. compared traditional and physicochemical approaches in evaluating acid–base disorders among ICU-admitted COVID-19 patients. Their findings highlighted the superior diagnostic accuracy of Stewart’s physicochemical method over traditional approaches, with a higher detection rate of hidden metabolic abnormalities. This approach may improve risk stratification and therapeutic decisions in critically ill populations (Contribution 5).

Laou et al. investigated the relationship between the calculated plasma volume status (cPVS), sublingual microcirculatory blood flow, and postoperative organ injury in surgical patients. Their findings indicated that a higher postoperative cPVS was significantly associated with an increased risk of organ complications, including sepsis and respiratory failure. This study underscores the potential role of cPVS monitoring in perioperative fluid management to mitigate postoperative risks (Contribution 6).

## 5. Impact of the Pandemic on Chronic Respiratory Diseases

The COVID-19 pandemic disrupted healthcare systems worldwide, significantly affecting the management of chronic respiratory diseases. Patients with conditions such as chronic obstructive pulmonary disease (COPD) experienced reduced access to routine care, leading to delays in treatment and increased disease severity [11]. While telemedicine has been proposed as a solution to bridge gaps in healthcare access, questions remain regarding its long-term efficacy in managing chronic respiratory conditions.

This Special Issue features work by Ahn and Park, who conducted a systematic review and meta-analysis comparing COPD hospitalization and mortality before and during the pandemic. Their findings suggest that while hospital admissions for COPD exacerbations declined during the pandemic, the in-hospital mortality increased significantly, highlighting the unintended consequences of reduced healthcare access (Contribution 7). These results underscore the importance of developing hybrid models of care that integrate both in-person and telemedicine-based approaches to ensure continuity in the management of chronic respiratory diseases.

## 6. Unusual Cases and New Clinical Insights in Emergency Medicine

While respiratory and emergency medicine research often focuses on large-scale public health concerns, rare and complex cases provide valuable insights into diagnostic challenges and treatment innovations.

Konstantinidou et al. presented a unique case of recurrent echinococcosis manifesting as hydatoptysis, illustrating the complexities of managing parasitic infections in patients with chronic respiratory disease (Contribution 8). Such cases emphasize the importance of improving diagnostic algorithms for rare conditions.

## 7. Conclusions

The research presented in this Special Issue reflects the dynamic nature of respiratory and emergency medicine, addressing key challenges while paving the way for future advancements. Despite significant progress in prognostic modeling, post-COVID-19 care, metabolic assessment, chronic disease management, and emergency diagnostics, substantial gaps remain. The continued integration of AI, personalized medicine, and standardized clinical protocols will be essential to advancing the field. By fostering interdisciplinary collaboration and leveraging emerging technologies, respiratory and emergency medicine can continue to evolve, ensuring better outcomes for critically ill and respiratory-compromised patients in the years to come.
